# Energy-Efficient Dynamic Workflow Scheduling in Cloud Environments Using Deep Learning

**DOI:** 10.3390/s25051428

**Published:** 2025-02-26

**Authors:** Sunera Chandrasiri, Dulani Meedeniya

**Affiliations:** Department of Computer Science and Engineering, University of Moratuwa, Moratuwa 10400, Sri Lanka; chandrasirikdsa.24@uom.lk

**Keywords:** artificial intelligence, cloud workflow scheduling, Graph Neural Network, multi-objective optimization, reinforcement learning

## Abstract

Dynamic workflow scheduling in cloud environments is a challenging task due to task dependencies, fluctuating workloads, resource variability, and the need to balance makespan and energy consumption. This study presents a novel scheduling framework that integrates Graph Neural Networks (GNNs) with Deep Reinforcement Learning (DRL) using the Proximal Policy Optimization (PPO) algorithm to achieve multi-objective optimization, focusing on minimizing makespan and reducing energy consumption. By leveraging GNNs to model task dependencies within workflows, the framework enables adaptive and informed resource allocation. The agent was evaluated within a CloudSim-based simulation environment using synthetic datasets. Experimental results across benchmark datasets demonstrate the proposed framework’s effectiveness, achieving consistent improvements in makespan and energy consumption over traditional heuristic methods. The framework achieved a minimum makespan of 689.22 s against the second best of 800.72 s in moderate-sized datasets, reducing makespan significantly with improvements up to 13.92% over baseline methods such as HEFT, Min–Min, and Max–Min, while maintaining competitive energy consumption of 10,964.45 J. These findings highlight the potential of combining GNNs and DRL for dynamic task scheduling in cloud environments, effectively balancing multiple objectives.

## 1. Introduction

Cloud computing has emerged as a transformative force in modern computing, providing scalable, versatile resources and driving significant technological advancements across industries [1]. According to the National Institute of Standards and Technology (NIST), cloud computing is defined as a model that enables easy and on-demand access to a shared pool of configurable computing resources—such as networks, servers, storage, applications, and services—that can be rapidly provisioned and released with minimal management or involvement from the service provider [2]. As cloud computing continues to evolve, deployment models such as public, private, community, and hybrid clouds offer tailored solutions to meet diverse organizational needs [2].

**Figure 1:**
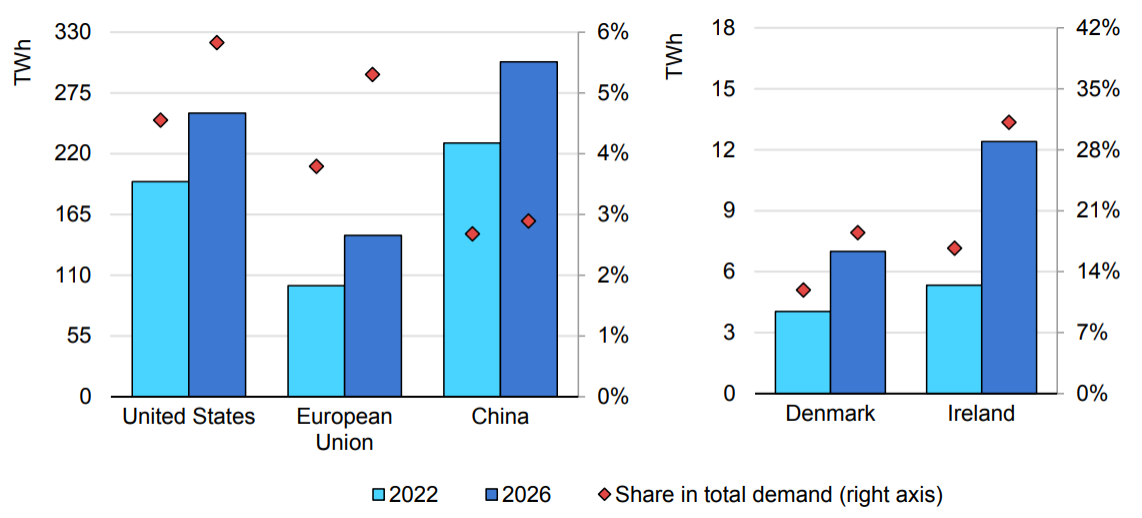
Electricity demand of data centers in the United States, European Union, and China (**left**) and Denmark and Ireland (**right**) for the years 2022 and 2026. The left axis represents total electricity consumption, while the right axis indicates their share in total electricity demand [4].

With these various offerings, the global cloud computing market is expanding rapidly with a Compound Annual Growth Rate (CAGR) of 21.2%. Market forecasts predict that the market, valued at USD 626.4 billion in 2023, will reach USD 1266.4 billion by 2028 [3]. However, this rapid growth comes at a cost, particularly in energy consumption within data centers, which are critical for cloud services. In 2022, data centers in the United States, European Union, and China accounted for significant electricity demand, with expectations for substantial growth across all regions by 2026 [4] (Figure 1).

Given this trend, minimizing power consumption has become a key concern in data centers [5]. Benchmarks on various CPUs indicate that power consumption increases with workloads, highlighting the potential for electricity savings through efficient server load management [6]. Approximately 45% of the energy use in the data center is directed toward IT equipment, such as CPUs and storage devices [5]. Optimizing energy efficiency through strategic resource allocation thus offers a practical solution to address these challenges.

Cloud architecture leverages virtualization, where a hypervisor divides a physical server into multiple isolated virtual machines (VMs). Each VM operates independently, running its own operating system and applications (tasks) as if it were a standalone server [7]. Within cloud environments, VMs handle tasks that span applications such as scientific workflows, batch processing, and distributed systems. The assignment of tasks in cloud environments is managed by a scheduler with the aim of optimizing this allocation. This problem, known as the cloud task-scheduling problem, is an NP-hard combinatorial optimization problem [8]. Given the exponential time complexity of NP-hard problems, numerous approximation algorithms such as heuristics, meta-heuristics, and machine learning techniques have been developed to produce feasible solutions within reasonable timeframes [9,10].

Scheduling approaches in cloud environments are generally categorized as either (1) static scheduling, which requires advanced knowledge of tasks and resources, or (2) dynamic scheduling, which requires adapting to changing workloads in different settings [10]. For example, static scheduling is well suited for predefined workflows, such as nightly batch jobs, where task attributes are known in advance, allowing tailored algorithms. On the other hand, dynamic scheduling is required for real-time allocation in cloud-hosted services where the exact characteristics of tasks is often remain unknown until execution. This paper focuses on the dynamic scheduling of dependent tasks, where tasks are modeled as workflows represented by Directed Acyclic Graphs (DAGs) [11]. DAG-based structures are widely implemented in workflow engines such as Apache Airflow, OpenStack Mistral, Argo Workflows, AWS Batch, and Azure Batch [12,13,14,15,16].

Cloud task schedulers typically aim to optimize specific objectives. Common objectives include minimizing makespan, reducing energy consumption, minimizing service-level agreement (SLA) violations, lowering costs, improving reliability, balancing loads, reducing response times, and maximizing throughput [10]. Among these, makespan is a primary metric for evaluating the effectiveness of workflow scheduling, representing the total time required to complete a set of tasks. An optimized schedule reduces makespan, enhancing the overall system performance. Additionally, energy consumption has become a critical focus, driven by the need to reduce power usage in energy-intensive data centers. The emphasis on energy efficiency in cloud computing aligns with the growing global imperative for sustainable technology practices. Data centers, which are at the core of cloud infrastructure, account for a significant portion of global energy consumption, contributing to substantial carbon emissions [17]. The global energy demand of data centers is projected to increase, driven by the expansion of cloud services and the rapid increase of data-intensive applications and Artificial Intelligence (AI) applications [18]. Consequently, optimizing energy usage has become a critical priority not only to reduce operational costs but also to meet sustainability goals outlined in global frameworks like the Paris Agreement [19]. This study adopts a multi-objective optimization framework for workflow scheduling, explicitly focusing on minimizing makespan and reducing energy consumption, which are critical for efficient and sustainable cloud performance.

**Figure 2:**
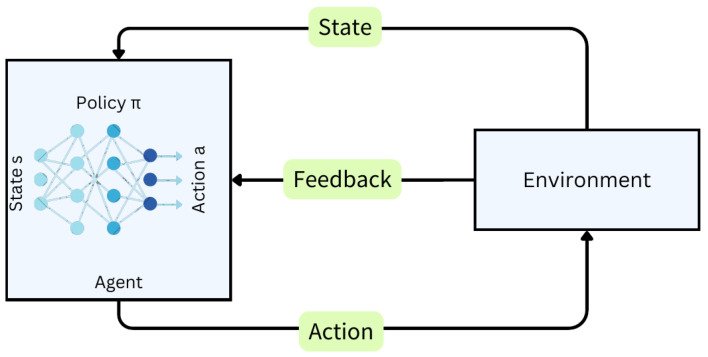
Interaction flow of a Reinforcement Learning framework. The agent observes the state *s* of the environment and selects an action *a* based on its policy π. The action influences the environment, resulting in feedback and a new state, enabling the agent to iteratively improve its decision-making process.

Deep Reinforcement Learning (DRL), a branch of machine learning that leverages deep neural networks, enables agents to learn optimal actions by interacting with their environment [20]. In DRL frameworks, an agent observes the current state, selects actions, and receives feedback in the form of rewards, which guide its learning toward long-term objectives (Figure 2). DRL has shown promise in addressing complex resource allocation problems, with several studies applying DRL techniques to the cloud task-scheduling problem [9]. However, most existing research focuses on independent tasks, leaving the application of DRL to dynamic workflows largely underexplored.

This gap can be attributed to the unique challenges of workflow scheduling, where tasks are interdependent and must follow precedence constraints. Unlike independent task scheduling, which focuses on assigning single tasks to VMs, workflow scheduling involves multiple layers of decision-making: selecting the workflow to process, determining the next executable task within it, and assigning the task to an appropriate VM. This additional complexity introduced by workflows challenges traditional approximation methods.

Recently, Graph Neural Networks (GNNs) have gained attention for their capability to capture dependencies in graph-structured data. While studies integrating GNNs with DRL for workflow scheduling in cloud environments remain limited, GNNs have demonstrated success in related fields such as the Flexible Job Shop Scheduling Problem, where they effectively model complex task dependencies [21,22,23]. This suggests that combining GNNs with DRL could enhance decision-making in dynamic workflow scheduling, enabling optimized schedules that respect task dependencies and execution constraints.

In this paper, we propose a novel framework for cloud workflow scheduling by leveraging DRL and GNNs. Our approach minimizes makespan while also improving energy efficiency. The main contributions of this article are listed as follows:

- Development of a novel DRL-GNN framework for dynamic workflow scheduling.
- Implementation of a multi-objective optimization strategy using immediate reward based on heuristics targeting makespan and energy consumption.
- Experimental evaluation against multiple heuristic algorithms with improvements over them.

To clarify the motivation of this work, we focus on addressing key dynamics in both workflow structures and cloud environments. Workflow dynamics include variations in task dependencies, sizes, and execution orders, modeled as DAGs that reflect diverse real-world workflows ranging from small, independent tasks to complex, interdependent structures. Cloud environment dynamics encompass variability in resource availability, such as fluctuating VM configurations and changing workloads. Unlike static algorithms like HEFT, which require prior knowledge of the entire task set before scheduling, the proposed model dynamically assigns tasks without knowing their specifics in advance, enabling real-time adaptability. By targeting these dynamic aspects, the framework provides scheduling solutions that optimize both makespan and energy consumption under varying conditions, addressing critical gaps in static or single-objective scheduling approaches.

The remainder of the paper is organized as follows: Section 2 provides a review of relevant literature. In Section 3, we present the problem formulation, feature mapping, and the proposed Reinforcement Learning framework. Section 4 discusses the evaluation methodology and results, including comparisons with related algorithms. Section 5 presents a comparison of proposed method with other related algorithms and outlines limitations and potential directions for future work. Finally, Section 6 presents the conclusions.

## 2. Related Studies

**Figure 3:**
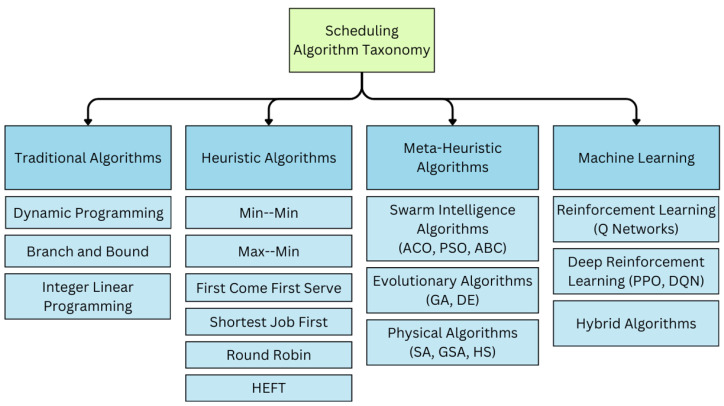
Scheduling algorithm taxonomy.

Over the years, a variety of scheduling algorithms have been developed for cloud task scheduling, as depicted in Figure 3. Traditional methods, such as Dynamic Programming, Branch and Bound, and Integer Linear Programming, focus on finding optimal solutions. However, these methods are often impractical for large-scale cloud environments due to the NP-hard nature of the cloud task-scheduling problem. To address this limitation, heuristic approaches like Min–Min, Max–Min, First Come First Serve (FCFS), Shortest Job First (SJF), Round Robin (RR), and Heterogeneous Earliest Finish Time (HEFT) offer computationally feasible, though approximate, solutions [24]. While these methods are efficient for scheduling, they have limited adaptability in dynamic cloud environments. For example, the Energy- and Performance-Efficient Task-Scheduling Algorithm (EPETS) [25] proposes a two-stage approach to reduce energy consumption and improve performance, but it primarily focuses on independent tasks and does not address dependencies within workflows.

Meta-heuristic algorithms provide greater adaptability and robustness in many scheduling scenarios. These include Swarm Intelligence Algorithms (e.g., Ant Colony Optimization, Particle Swarm Optimization, Artificial Bee Colony), Evolutionary Algorithms (e.g., Genetic Algorithms, Differential Evolution), and Physics-inspired Algorithms, such as the Gravitational Search Algorithm and Harmony Search [10,26]. Meta-heuristic approaches have emerged as powerful solutions for workflow scheduling in cloud environments, offering flexibility and robustness in addressing multi-objective optimization challenges. Techniques such as the Multi-objective Quantum-inspired Genetic Algorithm (MQGA) utilize innovative principles like qubits for chromosome representation and quantum rotation gates to enhance population diversity and convergence [27]. These methods have demonstrated significant improvements in energy consumption and makespan optimization, showcasing the potential of quantum-inspired approaches in hybrid cloud environments. Additionally, meta-heuristics based on NSGA-II [28,29] have been proposed to tackle multi-objective optimization problems in scheduling. Other innovative methods, such as CE-PRO [30], employ the Poor and Rich optimization algorithm, introducing novel heuristics for efficient resource allocation. However, these approaches are static scheduling algorithms, designed for predefined workflows and unable to dynamically adapt to evolving workloads or dependency-aware scenarios. Furthermore, they remain time-consuming because of the complexities of task dependencies and varying workloads as well as the iterative nature of such algorithms [21].

In recent years, machine learning techniques, particularly Reinforcement Learning (RL), have gained attention for cloud scheduling applications. RL approaches, including Policy Optimization and Q-Networks, have shown promise for dynamic environments Building on these foundations, Deep Reinforcement Learning (DRL) methods, such as Proximal Policy Optimization (PPO), Deep Q-Network (DQN), and Double Deep Q-Network (DDQN), bring advanced learning capabilities to complex scheduling problems [9].

Despite significant advancements in cloud scheduling, several limitations persist in the current research. Many DRL-based studies focus primarily on static scheduling or independent tasks, with limited exploration of dynamic, dependency-aware scheduling for cloud workflows. For example, DRL-Cloud [31] applies a deep Q-learning model for optimizing resource provisioning and task scheduling, focusing on reducing energy consumption and costs for tasks with dependencies. DRLBTSA [32] introduces dynamic task scheduling in a batch environment, optimizing energy consumption, SLA violations, and makespan. While DRLBTSA supports dynamic scheduling, it does not account for task dependencies and is limited to independent tasks. HunterPlus [33] further explores DRL-based approaches by integrating gated recurrent units (GRUs) and convolutional neural networks (CNNs) for independent task scheduling. However, HunterPlus is restricted to cloud-fog environments and does not address task dependencies.

In more recent work, DeepRM Plus [34] employs CNNs for state mapping to optimize turnaround time and cycling time for independent tasks, using imitation learning to enhance convergence. Other recent frameworks, such as MADRL [11] and DPSO-GA [35], focus on multi-objective optimization in cloud environments. MADRL leverages Multi-Agent Reinforcement Learning (MARL) to optimize green energy utilization in multi-cloud settings. DPSO-GA combines DRL with meta-heuristics, specifically PSO and GA, to optimize waiting time, task migration, and response time. However, PSO-GA does not support task dependencies. Several studies have focused on optimizing workflow scheduling in geographically distributed cloud data centers. These approaches often address challenges related to varying electricity prices and data transmission times across data centers. For example, DEWS (Deadline-constrained Energy-aware Workflow Scheduling) incorporates Dynamic Voltage Frequency Scaling (DVFS) to minimize energy costs while meeting task deadlines by leveraging differences in electricity prices [36]. Similarly, other algorithms consider the trade-offs between data transmission costs and energy consumption to enhance efficiency in distributed cloud environments [37]. While these methods provide effective solutions for geographically distributed setups, they primarily focus on static constraints and energy-aware strategies without dynamically learning from task dependencies.

Beyond these, the Priority-Aware Deployment Framework for Service Function Chains (SFCs) [38] leverages a deep reinforcement learning algorithm for multi-objective optimization, prioritizing latency and cost efficiency in NFV-based networks. Unlike DRL-GNN, which focuses on task dependencies and cloud workflow scheduling, this framework is specifically tailored to dynamic network services and real-time deployment challenges. Similarly, the Adaptive Workload Management Model [39] employs queuing theory to enhance workload balancing and SLA compliance in dynamic cloud environments. While it effectively manages resource optimization, it does not incorporate deep learning or graph-based modeling to address dependency-aware scheduling challenges.

The Flexible Job Shop Scheduling Problem (FJSSP) in Operational Research shares several similarities with the cloud task-scheduling problem, as both involve allocating a set of tasks or jobs to a limited pool of resources (machines in FJSSP and VMs in cloud scheduling) under specific constraints. In FJSSP, each job consists of a series of operations, with each operation capable of being executed on one or more machines. This is similar to cloud scheduling, where tasks can be assigned to multiple VMs based on resource compatibility. Constraints play a crucial role in both problems: FJSSP considers machine capabilities and limitations, much like cloud scheduling, which must consider VM requirements such as RAM, storage, and bandwidth. However, a key difference is that cloud workflows are often represented by DAGs, while FJSSP typically employs simpler precedence constraints. Given these parallels, there is potential to explore whether approaches developed for FJSSP could be adapted for cloud scheduling. Several FJSSP algorithms that utilize GNNs have shown promising results [40]. For instance, in [21], a GNN-based approach with a multi-action setup demonstrated significant improvements over existing methods. Additionally, ref. [41] presents a solution for the Dynamic FJSSP (DFJSSP) with random task arrivals using hierarchical schedulers, where a higher-level agent releases tasks to a lower-level agent that leverages GNNs for efficient scheduling.

In cloud computing, integrating GNNs with DRL for workflow scheduling remains largely unexplored, even though GNNs have significant potential to capture complex task dependencies and enhance decision-making, as demonstrated via FJSSP. While scheduling approaches in FJSSP successfully leverage GNNs, these methods have not been adapted to the unique requirements of cloud computing, including dynamic workload fluctuations and energy efficiency considerations. This highlights a clear opportunity for innovation by exploring GNNs within DRL frameworks to capture task dependencies more effectively.

Moreover, DRL solutions that explore multi-objective optimization with regard to makespan and energy efficiency remains largely unexplored in cloud environments with dependent tasks. This gap underscores the need for approaches capable of balancing these objectives while respecting task dependencies and dynamic cloud conditions.

The proposed study aims to bridge these gaps by encoding task dependencies using GNNs and integrating them with a DRL framework to dynamically optimize scheduling policies. This approach not only addresses precedence constraints and interdependencies but also demonstrates scalability across varying workflow complexities. By incorporating multi-objective optimization, the study seeks to address the dual challenges of reducing makespan and improving energy efficiency, thus addressing limitations in current research and advancing cloud workflow scheduling.

## 3. Solution Design and Development

**Figure 4:**
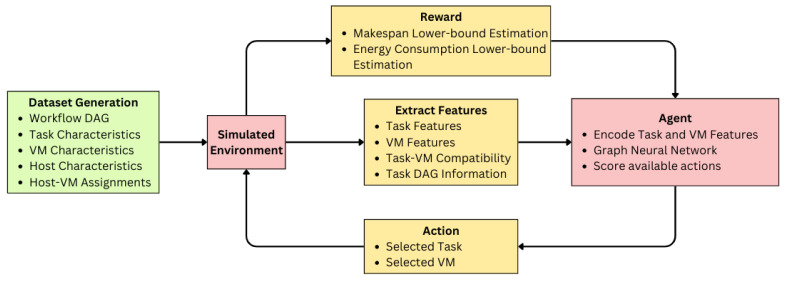
RL methodology used for the proposed algorithm. The agent will train with the PPO algorithm using the reward and features from the simulated environment that changes according to the agent actions.

Figure 4 illustrates the architectural overview of the Reinforcement Learning methodology used in this study. Initially, datasets are generated based on various parametric configurations. A simulated environment serves as the foundation for the training process, wherein the agent interacts with the environment by executing actions and receiving corresponding rewards along with state information and environmental features. Through this feedback mechanism, the agent learns to optimize the dual objectives: minimizing makespan and reducing energy consumption.

The subsequent sections are structured as follows. The formal model formulation that underpins both dataset generation and the simulated environment is first presented. Lower-bound heuristics, which play a crucial role in reward formulation, are then introduced. This is followed by a discussion of the feature extraction process employed. Finally, the agent architecture and the Proximal Policy Optimization (PPO) training procedure are elaborated, with particular emphasis on the integration of Graph Neural Networks, specifically the Graph Isomorphism Network (GIN).

### 3.1. Workflow Modeling

**Figure 5:**
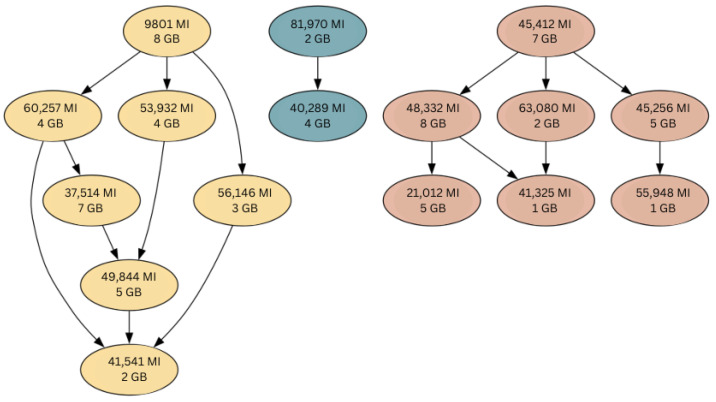
Sample workflows represented as DAGs. Each workflow consists of tasks (nodes) linked by directed edges, which indicate the dependencies between tasks, with tasks annotated with their computational requirement (in Million Instructions—MI) and memory usage (in Gigabytes—GB).

In this study, workflows are represented as Directed Acyclic Graphs (DAGs), which are commonly used to model dependencies between tasks [31]. A DAG is an acyclic graph G=(V,E), where *V* is the set of vertices (tasks) and E⊆V×V is the set of directed edges representing dependencies between tasks. For any directed edge (u,v)∈E, task *u* must be completed before task *v* can start, as shown in Figure 5.

Makespan is a key metric in scheduling workflows, representing the total time required to complete all tasks in a workflow. Given a DAG G=(V,E) representing the workflow, the makespan *M* of the workflow is defined as in Equation (1). Here, $M_{J_{i}}$ denotes the completion time of task $J_{i}\in V$. The value of $M_{J_{i}}$ can be derived as shown in Equation (2). In this expression, $t^{\prime }$ represents the time at which the scheduler assigns task $J_{i}$ to ${\mathrm{VM}}_{k}$, and $T_{J_{i},{\mathrm{VM}}_{k}}$ is the execution time of task $J_{i}$ on ${\mathrm{VM}}_{k}$. For this scheduling to occur, the task and VM must be compatible.

(1) $$M=\max_{J_{i}\in V}M_{J_{i}}$$

(2) $$M_{J_{i}}=t^{\prime }+T_{J_{i},{\mathrm{VM}}_{k}}$$

Compatibility is determined by the memory requirements of the task and the VM. A task $J_{i}$ can only be assigned to ${\mathrm{VM}}_{j}$ if $R_{J_{i}}\leq R_{{\mathrm{VM}}_{j}}$, where $R_{J_{i}}$ is the memory required by task $J_{i}$, and $R_{{\mathrm{VM}}_{j}}$ is the memory allocated to ${\mathrm{VM}}_{j}$. When this condition is satisfied, the execution time $T_{J_{i},{\mathrm{VM}}_{j}}$ can be calculated using Equation (3). Here, $L_{J_{i}}$ is the length of task $J_{i}$, and $S_{{\mathrm{VM}}_{j}}$ is the CPU speed of ${\mathrm{VM}}_{j}$. It is assumed that the VM operates at full utilization with a constant CPU speed $S_{{\mathrm{VM}}_{j}}$ during the execution of the task.

(3) $$T_{J_{i},{\mathrm{VM}}_{j}}=\frac{L_{J_{i}}}{S_{{\mathrm{VM}}_{j}}}$$

Total energy consumption is a critical metric in cloud scheduling, representing the cumulative energy used by all hosts in a data center over a given period. The total energy consumption, denoted as $E^{\mathrm{total}}$, is defined as in Equation (4). Here, $P^{\mathrm{total}}\left(t\right)$ represents the total power consumption of the data center at time *t*. It is calculated as the sum of the power consumption of all $N^{H}$ hosts, as shown in Equation (5).

(4) $$E^{\mathrm{total}}=\int_{0}^{T}P^{\mathrm{total}}\left(t\right)\;dt$$

(5) $$P^{\mathrm{total}}\left(t\right)=\sum_{i=1}^{N^{H}}P_{H_{i}}\left(t\right)$$

The power consumption $P_{H_{i}}\left(t\right)$ of host $H_{i}$ at time *t* is determined by its utilization $U_{H_{i}}\left(t\right)$, which varies between the thresholds $U_{H_{i}}^{\mathrm{low}}$ and $U_{H_{i}}^{\mathrm{high}}$. The corresponding power values at these thresholds are $P_{H_{i}}^{\mathrm{low}}$ and $P_{H_{i}}^{\mathrm{high}}$. Assuming a linear relationship between utilization and power, $P_{H_{i}}\left(t\right)$ can be expressed as in Equation (6). For simplicity, using the power at idle ($P_{H_{i}}^{\mathrm{idle}}$) and peak utilization ($P_{H_{i}}^{\mathrm{peak}}$), the above equation can be reduced to Equation (7). The utilization $U_{H_{i}}\left(t\right)$ of host $H_{i}$ is influenced by the VMs running tasks on it. Let ${\mathrm{VM}}_{H_{i},t}^{\mathrm{active}}$ represent the active VMs on host $H_{i}$ at time *t*. The utilization $U_{H_{i}}\left(t\right)$ is given by Equation (8). Here, $S_{{\mathrm{VM}}_{j}}$ is the CPU speed of ${\mathrm{VM}}_{j}$, and $S_{H_{i}}$ is the CPU speed of host $H_{i}$.

(6) $$P_{H_{i}}\left(t\right)=P_{H_{i}}^{\mathrm{low}}+\left(P_{H_{i}}^{\mathrm{high}}-P_{H_{i}}^{\mathrm{low}}\right)\cdot \frac{U_{H_{i}}\left(t\right)-U_{H_{i}}^{\mathrm{low}}}{U_{H_{i}}^{\mathrm{high}}-U_{H_{i}}^{\mathrm{low}}}$$

(7) $$P_{H_{i}}\left(t\right)=P_{H_{i}}^{\mathrm{idle}}+\left(P_{H_{i}}^{\mathrm{peak}}-P_{H_{i}}^{\mathrm{idle}}\right)\cdot U_{H_{i}}\left(t\right)$$

(8) $$U_{H_{i}}\left(t\right)=\sum_{{\mathrm{VM}}_{j}\in {\mathrm{VM}}_{H_{i},t}^{\mathrm{active}}}\frac{S_{{\mathrm{VM}}_{j}}}{S_{H_{i}}}$$

The objective is to find an optimal scheduling policy that minimizes both makespan (*M*) and total energy consumption ($E^{\mathrm{total}}$). This multi-objective optimization problem is formulated as shown in Equation (9). The optimization is subject to the constraints:

- Tasks must satisfy memory requirements, i.e., $R_{J_{i}}\leq R_{{\mathrm{VM}}_{j}}$.
- Precedence constraints defined by a DAG must be respected.

(9) $$\mathrm{minimize}\;\left(M,E^{\mathrm{total}}\right)$$

### 3.2. Lower-Bound Heuristics

In this study, lower-bound heuristics provide baseline estimations for makespan and energy consumption, which are later used to formulate the reward function. These lower bounds serve as optimistic estimates, ensuring that the actual makespan and energy consumption will always be greater than or equal to these values. The makespan lower-bound estimation at time *t* is calculated by determining the maximum estimated completion time among all tasks in the workflow DAG. For each task $J_{i}$, the completion time lower bound depends on its scheduling status. If a task is scheduled, its actual completion time is used. For unscheduled tasks, the completion time lower bound is computed by evaluating all compatible VMs and considering both parent task availability and the VM’s readiness.

Formally, the makespan lower-bound estimation, denoted as $M_{t}^{\mathrm{est}}$, is defined as shown in Equation (10). Here, $M_{J_{i},t}^{\mathrm{est}}$ represents the estimated completion time lower bound of task $J_{i}$. This is recursively derived using Equation (11), where ${\mathrm{VM}}_{J_{i}}^{\mathrm{cmp}}$ represents the set of compatible VMs for task $J_{i}$, $J_{J_{i}}^{\mathrm{par}}$ denotes the parent tasks of $J_{i}$, and $M_{{\mathrm{VM}}_{j},t}$ is the current finish time of ${\mathrm{VM}}_{j}$, as defined in Equation (12). Here, $J_{{\mathrm{VM}}_{j},t}^{sch}$ refers to all the tasks scheduled on ${\mathrm{VM}}_{j}$ before time *t*, and $M_{J_{k}}$ is the completion time of task $J_{k}$, as defined in Equation (2).

(10) $$M_{t}^{\mathrm{est}}=\max_{J_{i}\in V}M_{J_{i},t}^{\mathrm{est}}$$

(11) $$M_{J_{i},t}^{\mathrm{est}}=\left\{\begin{array}{cc} M_{J_{i}} & \mathrm{if}\;J_{i}\;\mathrm{is}\;\mathrm{already}\;\mathrm{scheduled},\\ \min_{{\mathrm{VM}}_{j}\in {\mathrm{VM}}_{J_{i}}^{\mathrm{cmp}}}\left(\max_{J_{k}\in J_{J_{i}}^{\mathrm{par}}}\left(M_{J_{k},t}^{\mathrm{est}},M_{{\mathrm{VM}}_{j},t}\right)+T_{J_{i},{\mathrm{VM}}_{j}}\right) & \mathrm{otherwise}. \end{array}\right.$$

(12) $$M_{{\mathrm{VM}}_{j},t}=\max_{J_{k}\in J_{{\mathrm{VM}}_{j},t}^{sch}}M_{J_{k}}$$

The active energy consumption lower-bound estimation at time *t* is computed using the energy consumption explicitly caused by running jobs (disregarding the idle power consumption). If a task is scheduled, its active energy consumption is based on the task’s execution time and the assigned VM. For unscheduled tasks, the active energy consumption is estimated as the minimum possible energy cost across compatible VMs. Formally, the active energy consumption lower-bound estimation, denoted as $E_{t}^{\mathrm{est}}$, is defined in Equation (13). Here, *V* represents all the tasks, and $E_{J_{i},t}^{\mathrm{est}}$ is the estimated active energy cost of task $J_{i}$. This cost is defined in Equation (14), where In $E_{J_{i},{\mathrm{VM}}_{j}}$ is the active energy consumed when task $J_{i}$ is scheduled on ${\mathrm{VM}}_{j}$. It is calculated using Equation (15), where $T_{J_{i},{\mathrm{VM}}_{j}}$ is the execution time of task $J_{i}$ on ${\mathrm{VM}}_{j}$, and $P_{{\mathrm{VM}}_{j}}$ is the power consumption of ${\mathrm{VM}}_{j}$. The power consumption $P_{{\mathrm{VM}}_{j}}$ is derived as shown in Equation (16) using Equation (8) and the active component of Equation (7). Here, $P_{H_{k}}^{\mathrm{peak}}$ and $P_{H_{k}}^{\mathrm{idle}}$ represent the peak and idle power consumption of the host $H_{k}$ where ${\mathrm{VM}}_{j}$ is located. The term $S_{{\mathrm{VM}}_{j}}$ is the CPU speed of ${\mathrm{VM}}_{j}$, and $S_{H_{k}}$ is the CPU speed of the host $H_{k}$.

(13) $$E_{t}^{\mathrm{est}}=\sum_{J_{i}\in V}E_{J_{i},t}^{\mathrm{est}}$$

(14) $$E_{J_{i},t}^{\mathrm{est}}=\left\{\begin{array}{cc} E_{J_{i},{\mathrm{VM}}_{k}}, & \mathrm{if}\;J_{i}\;\mathrm{is}\;\mathrm{already}\;\mathrm{scheduled}\;\mathrm{to}\;{\mathrm{VM}}_{k},\\ \min_{{\mathrm{VM}}_{j}\in {\mathrm{VM}}_{J_{i}}^{\mathrm{cmp}}}\left(E_{J_{i},{\mathrm{VM}}_{j}}\right), & \mathrm{otherwise}. \end{array}\right.$$

(15) $$E_{J_{i},{\mathrm{VM}}_{j}}=T_{J_{i},{\mathrm{VM}}_{j}}\cdot P_{{\mathrm{VM}}_{j}}$$

(16) $$P_{{\mathrm{VM}}_{j}}=\left(P_{H_{k}}^{\mathrm{peak}}-P_{H_{k}}^{\mathrm{idle}}\right)\cdot \frac{S_{{\mathrm{VM}}_{j}}}{S_{H_{k}}}$$

### 3.3. Data Preprocessing

To ensure faster convergence of the model, the input data are preprocessed into a structured format suitable for the Reinforcement Learning framework. The preprocessing step handles task-level, VM-level, and pairwise information, as well as the task dependency graph. Input feature information is given in Table 1. Additionally, since a workflow may contain multiple start and end nodes, the algorithm adds a common start node and end node consisting of dummy tasks (with 0 MI length and compatibility with all VMs). This is helpful in simplifying the workflows.

### 3.4. Reinforcement Learning Framework

Reinforcement Learning (RL) is a paradigm in machine learning in which an agent interacts with an environment to learn optimal actions through trial and error. The agent receives feedback in the form of rewards, which guide its learning to maximize cumulative rewards over time.

- State (*s*): This represents the environment’s current condition. The state provides essential information for the agent to make decisions. Let $S_{t}$ denote the state at time *t*.
- Action (*a*): An action is a choice made by the agent in a given state. Let $A_{t}$ denote the action taken by the agent at time *t*.
- Reward (*r*): A scalar feedback signal provided by the environment after each action, indicating the immediate benefit of the action. The goal is to maximize the cumulative reward over time. Let $R_{t+1}$ be the reward received after taking action $A_{t}$ in state $S_{t}$.
- Policy (π): This defines the agent’s behavior by mapping states to probabilities of selecting each possible action. The policy can be deterministic (π(s)=a) or stochastic $\left(\pi \left(a|s\right)=P\left(A_{t}=a|S_{t}=s\right)\right)$.
- Value Function (V(s)): This estimates the expected cumulative reward an agent can obtain from a given state *s*, following a policy π. The value function is defined as in Equation (17) [42] where γ∈[0,1) is the discount factor, which balances immediate and future rewards.
  (17) $$V^{\pi }\left(s\right)=E_{\pi }\left[\sum_{k=0}^{\infty }\gamma^{k}R_{t+k+1}|S_{t}=s\right]$$
- Action-Value Function (Q(s,a)): This estimates the expected cumulative reward of taking an action *a* in state *s* and then following policy π. The action-value function is defined as in Equation (18) [42].
  (18) $$Q^{\pi }\left(s,a\right)=E_{\pi }\left[\sum_{k=0}^{\infty }\gamma^{k}R_{t+k+1}|S_{t}=s,A_{t}=a\right]$$

In DRL literature, Proximal Policy Optimization (PPO) is a popular algorithm designed to improve the stability and efficiency of policy updates in continuous and discrete action spaces [43]. PPO optimizes a policy by iteratively updating it using a clipped surrogate objective, which prevents large policy updates and ensures stable learning. The objective function in PPO is defined as in Equation (19) [43], where:

- $r_{t}\left(\theta \right)=\frac{\pi_{\theta }\left(a_{t}|s_{t}\right)}{\pi_{\theta_{\mathrm{old}}}\left(a_{t}|s_{t}\right)}$ is the probability ratio of the new policy $\pi_{\theta }$ to the old policy $\pi_{\theta_{\mathrm{old}}}$.
- ${\hat{A}}_{t}$ is the advantage estimate at time *t*, calculated as ${\hat{A}}_{t}=Q\left(s_{t},a_{t}\right)-V\left(s_{t}\right)$.
- ϵ is a hyperparameter that controls the range for clipping, limiting the extent of policy updates.

(19) $$L^{\mathrm{PPO}}\left(\theta \right)=E_{t}\left[\min\left(r_{t}\left(\theta \right){\hat{A}}_{t},\mathrm{clip}\left(r_{t}\left(\theta \right),1-\epsilon ,1+\epsilon \right){\hat{A}}_{t}\right)\right]$$

In this research, PPO is applied to the cloud task-scheduling problem, where the agent’s goal is to minimize both the makespan and energy consumption. The reward $R_{t}$ is designed to reflect the multi-objective optimization framework’s focus, penalizing the agent for longer makespans and higher energy usage. This dual-objective reward structure ensures that the agent learns to balance performance and sustainability in cloud environments effectively. To normalize the makespan and energy consumption, a normalization method based on time and cost estimations is employed. The reward function is given in Equation (20) where $M_{t}^{\mathrm{est}}$ and $E_{t}^{\mathrm{est}}$ are the makespan lower-bound and active energy consumption lower-bound estimations for the state $S_{t}$ (Equations (10) and (13)). Parameters α and β balance the importance of minimizing makespan and energy consumption.

(20) $$R_{t+1}=-\alpha \left(\frac{M_{t+1}^{\mathrm{est}}-M_{t}^{\mathrm{est}}}{M_{t+1}^{\mathrm{est}}}\right)-\beta \left(\frac{E_{t+1}^{\mathrm{est}}-E_{t}^{\mathrm{est}}}{E_{t+1}^{\mathrm{est}}}\right)$$

### 3.5. Agent Architecture and Model Design

**Figure 6:**
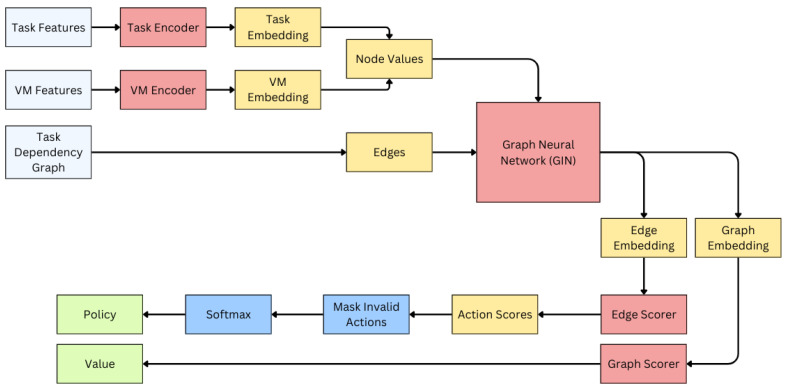
Agent architecture illustrating the encoding of tasks, VMs, and task–VM relationships, graph construction, GIN layers, and the action scoring mechanism. Red color blocks represent neural networks.

The agent is designed to optimize task scheduling by encoding task and VM features using a Graph Neural Network (GNN) architecture. The model comprises several components, including encoders for tasks and VMs pairs, followed by graph construction and processing through Graph Isomorphism Network (GIN) layers. The architecture is illustrated in Figure 6.

Each task is represented by a feature vector containing three elements: the task-scheduled flag, the task-ready flag, and the task completion time. These features are processed by the task encoder to generate task embeddings. Similarly, each VM is characterized by three features, the VM finish time, VM CPU speed, and VM active energy consumption rate, and is processed by the VM encoder to generate VM embeddings.

**Figure 7:**
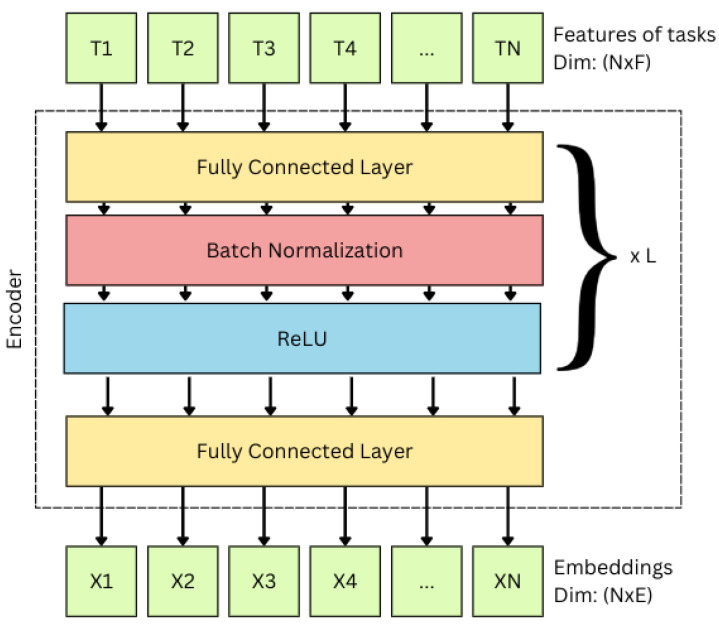
Structure of the encoder, including fully connected layers, batch normalization, and the transformation of features into embeddings.

The primary objective of these encoders is to transform task and VM features into a unified embedding space, enabling graph construction. Each encoder follows the same structure, as illustrated in Figure 7. The encoder is implemented as a Multi-Layer Perceptron (MLP) with batch normalization and ReLU activation. The MLP transformation for a single layer is defined as in Equation (21), where *x* is the input feature vector, $W^{\left(l\right)}$ and $b^{\left(l\right)}$ are the weights and biases of the *l*-th layer, and $z^{\left(l\right)}$ is the pre-activation output. Batch normalization is then applied to normalize $z^{\left(l\right)}$ as in Equation (22), where $\mu^{\left(l\right)}$ and $\sigma^{(l)2}$ are the mean and variance of $z^{\left(l\right)}$, and ϵ is a small constant for numerical stability. The normalized output is scaled and shifted using learnable parameters $\gamma^{\left(l\right)}$ and $\beta^{\left(l\right)}$. Finally, a ReLU activation function is applied following Equation (24) where $h^{\left(l\right)}$ is the output of the layer. This process is repeated for each layer in the MLP except for the last layer, in which only the MLP layer is applied.

(21) $$z^{\left(l\right)}=W^{\left(l\right)}x+b^{\left(l\right)}$$

(22) $${\hat{z}}^{\left(l\right)}=\frac{z^{\left(l\right)}-\mu^{\left(l\right)}}{\sqrt{\sigma^{(l)2}+\epsilon }}$$

(23) $$z^{\prime (l)}=\gamma^{\left(l\right)}{\hat{z}}^{\left(l\right)}+\beta^{\left(l\right)}$$

(24) $$h^{\left(l\right)}=\mathrm{ReLU}\left(z^{\prime (l)}\right)=\max\left(0,z^{\prime (l)}\right)$$

**Figure 8:**
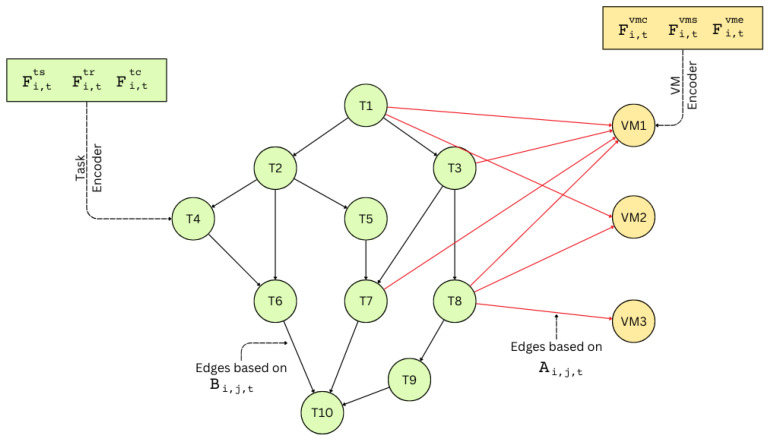
An example of a graph constructed from a workflow consisting of 10 tasks and 3 VMs. The green nodes represent task nodes, while the yellow nodes represent VM nodes. Edges between task nodes illustrate the dependency DAG, capturing task precedence relationships. The red edges indicate connections between task nodes and VM nodes, representing compatibility between tasks and VMs for scheduling.

During the graph construction phase, a heterogeneous graph is created where the nodes represent tasks and VMs, with their respective embeddings serving as the node features (Figure 8). The edges in the graph define the relationships between these nodes. For task–task edges, the adjacency matrix $A_{J_{i},J_{j},t}$ is used to derive the dependencies in the DAG, connecting tasks based on their precedence constraints. Edges between task nodes and VM nodes represent the assignability of tasks to VMs and are derived from the compatibility matrix $I_{J_{i},{\mathrm{VM}}_{j},t}^{\mathrm{cmp}}$. Thus, an edge $\left(J_{i},{\mathrm{VM}}_{j}\right)$ exists only if task $J_{i}$ is compatible with ${\mathrm{VM}}_{j}$. There are no edges inter-connecting VM nodes directly.

The constructed graph is processed through several Graph Isomorphism Network (GIN) layers [44], which update the node embeddings by aggregating information from neighboring nodes. Let $h_{v}^{\left(k\right)}$ denote the embedding of node *v* at the *k*-th layer, then $h_{v}^{\left(k\right)}$ can be derived from the equation shown in Equation (25) where N(v) is the set of neighbors of node *v*, and $\epsilon^{\left(k\right)}$ is a learnable parameter. The overall graph embedding $h_{G}$ is obtained using mean pooling over all node embeddings in the last layer *L* as in Equation (26) where *N* is the number of nodes in the graph ($N_{J}+N_{\mathrm{VM}}$).

(25) $$h_{v}^{\left(k\right)}={\mathrm{MLP}}^{\left(k\right)}\left(\left(1+\epsilon^{\left(k\right)}\right)\cdot h_{v}^{\left(k-1\right)}+\sum_{u\in N(v)}h_{u}^{\left(k-1\right)}\right)$$

(26) $$h_{G}=\frac{1}{N}\sum_{i=1}^{N}h_{v_{i}}^{\left(L\right)}$$

After the GIN layers, the updated task node embeddings $h_{J_{i}}$ and VM node embeddings $h_{{\mathrm{VM}}_{j}}$ are extracted from the embedding of the last layer *L*. Then, for all compatible task–VM pairs, the action embeddings are created by concatenating the corresponding task node embedding, VM node embedding, and the graph embedding by following Equation (27). These action embeddings are passed through graph scorer, which is an MLP similar to the encoders but without batch normalization, to produce a scalar score $s_{J_{i},{\mathrm{VM}}_{j}}$ for each action.

(27) $$h_{J_{i},{\mathrm{VM}}_{j}}=h_{J_{i}}\;||\;h_{{\mathrm{VM}}_{j}}\;\left|\right|\;h_{G}$$

An action matrix $S\in R^{N\times M}$ is formed, where invalid actions (incompatible task–VM pairs) are assigned −∞ to mask them, and valid actions are assigned their corresponding scores. The policy is then obtained by applying the softmax function over the action matrix as in Equation (28) where A is the set of valid actions.

(28) $$\pi \left(J_{i},{\mathrm{VM}}_{j}\right)=\frac{\exp(s_{J_{i},{\mathrm{VM}}_{j}})}{\sum_{k,l\in A}\exp\left(s_{J_{k},{\mathrm{VM}}_{l}}\right)}$$

The above architecture is the architecture of the actor network. The actor network is responsible for generating a policy by determining the probabilities of assigning tasks to VMs. The critic network, on the other hand, is responsible for evaluating the current state by estimating the expected cumulative reward. It shares the same architecture as the actor network up to the GIN layers. The graph embedding $h_{G}$, obtained after mean pooling from the GIN layers, is passed through a value scorer, an MLP without batch normalization, to produce a scalar value estimation. This value represents the expected cumulative reward from the state $s_{t}$.

## 4. Result Evaluation

### 4.1. Dataset Generation

Each workflow is represented as a DAG generated using the G(n,p) method, where *n* is the number of nodes and *p* is the probability of creating an edge between any two nodes. The probability *p* is defined as $\frac{\log(n+\epsilon )}{n}$, where ϵ is a small constant to ensure a high probability of avoiding isolated vertices [45]. The number of nodes *n* in each generated DAG is set to $N^{J}-1$, and a start node is added to ensure the DAG remains connected.

The computational workload of each task, represented as its length, is constrained to the range $\left[L^{\min},L^{\max}\right]$. Task lengths are sampled from a normal distribution with a mean of $\frac{L^{\min}+L^{\max}}{2}$ and a standard deviation of $\frac{L^{\max}-L^{\min}}{6}$, ensuring most values fall within the specified range. Additionally, datasets with left-skewed and right-skewed distributions were generated to explore different task distributions and their impact on scheduling. These skewed distributions were created using skew-normal distributions, where left-skewed datasets prioritize shorter tasks and right-skewed datasets favor longer tasks, with the skewness (α) controlled around the same mean and standard deviation as the normal distribution. For this study, α=−5 was used to generate left-skewed distributions, while α=5 was used to generate right-skewed distributions, ensuring a substantial skewness. This variety in datasets provides a comprehensive evaluation of the scheduling method under diverse task workload scenarios.

For each dataset, the number of VMs is set to $N^{\mathrm{VM}}$, and the number of hosts to $N^{H}$. VMs are assigned to hosts randomly. Each VM’s allocated memory is chosen randomly between $R^{\min}$ and $R^{\max}$, while the memory requirements of tasks are capped to ensure compatibility with VM capacities. The CPU speeds for each VM is randomly set between $S_{\mathrm{VM}}^{\min}$ and $S_{\mathrm{VM}}^{\max}$. Host characteristics, such as CPU speed and power consumption, are derived from the SPECpower benchmark suite and listed in Table 2. The benchmark results originate from Dell (Round Rock, TX, USA) and IBM (Armonk, NY, USA). These publicly available performance metrics were utilized to simulate realistic energy consumption and processing capabilities in the experimental setup.

The number of workflows in each dataset is randomly chosen between 1 and $N^{W}$, creating varied levels of task concurrency.

### 4.2. Agent Training

To train the RL agent, a dataset was generated with the parameters shown in Table 3.

The training of the Reinforcement Learning agent was carried out using the PPO algorithm. The implementation was carried out in Python 3.12.7 following the CleanRL implementation [46]. The training was conducted over a fixed number of episodes, where each episode representing a complete scheduling scenario. At the end of each episode, the agent’s policy was updated using PPO to balance exploration and exploitation. The hyperparameters used for training the agent are summarized in Table 4.

**Figure 9:**
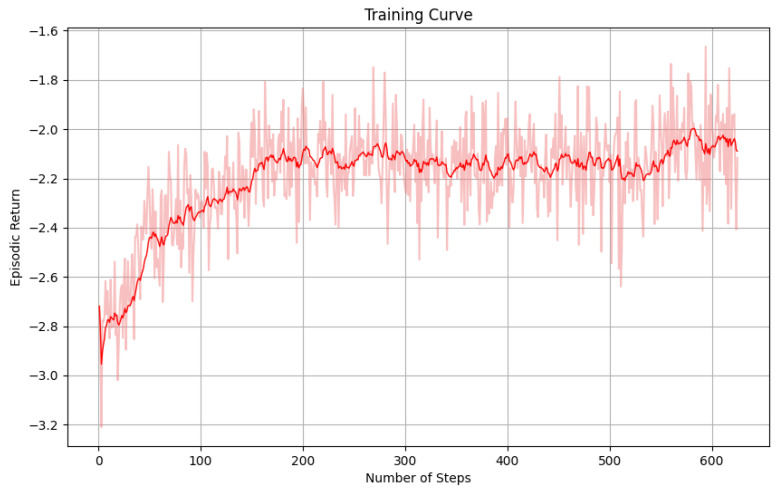
Episodic return during training, showing the agent’s performance improvement over time.

Figure 9 illustrates the learning curve of the agent, showing the increase in episodic return as training progressed. The training results indicate that the PPO algorithm, in conjunction with the GIN-based architecture, effectively learned to minimize the makespan and energy consumption while handling dynamic task arrivals and dependencies.

### 4.3. Cloud Computing Simulation Model

The evaluation of the proposed algorithm is conducted in a simulated cloud environment. Most research on cloud task and cloud workflow scheduling has utilized CloudSim [47], a widely used Java-based, open-source tool for simulating cloud environments [11,32,35,48]. CloudSim provides a flexible and extensible framework specifically designed for modeling and simulating cloud data centers and resource management strategies.

The proposed simulation model represents a multi-layered cloud computing environment designed to capture workflows, task dependencies, and resource dynamics. The architecture consists of multiple components, including data centers, VMs, and cloudlets (tasks), where each workflow is represented as a DAG of tasks. The Cloud Information Service (CIS) maintains a registry of all available resources across data centers, including VM configurations and their current states.

**Figure 10:**
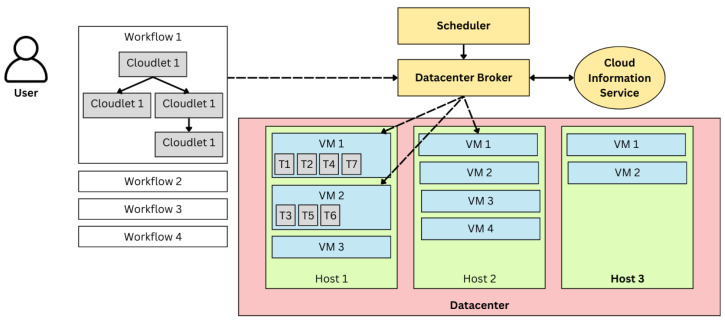
Cloud computing infrastructure and components assumed in the model architecture used for simulation developed in CloudSim.

The Data Center Broker acts as the intermediary between the user-submitted workflows and the underlying resources. The broker uses the implemented scheduler to dynamically allocate tasks to VMs. As depicted in Figure 10, tasks within workflows are dynamically assigned to VMs allocated across multiple hosts. This setup effectively emulates real-world scenarios where tasks compete for resources in heterogeneous environments.

The simulation supports various configurations, including the number of hosts and VMs. Workflow tasks are characterized by computational requirements (measured in Million Instructions) and memory needs, while VMs are configured with specific CPU speeds, RAM allocations, and energy consumption models. These configurations are based on parameters derived from real-world datasets and the SPECpower benchmark [6], ensuring heterogeneity in the simulation environment.

### 4.4. Agent Evaluation

For evaluating the RL agent, a dataset was generated with attributes given in Table 5. During evaluation, the number of workflows and tasks is varied to assess the model’s performance across several scenarios.

The current configuration of the CloudSim environment provides a controlled and manageable setup for evaluating the proposed algorithm under a variety of conditions. This configuration is designed to balance computational feasibility with the ability to assess the algorithm’s performance across diverse scenarios. By focusing on small- to medium-scale environments, the evaluation ensures clear insights into the algorithm’s capabilities, particularly for foundational testing and validation. For instance, in $DS_{3}$, tasks in the dataset are generated with lengths ranging from 500 to 100,000 MI, sampled from a normal distribution with a mean and a standard deviation calculated as described in Section 4.1. Workflows are modeled as DAGs of 40–50 tasks per workflow, and task dependencies are determined using an edge probability of $\frac{\log(n+\epsilon )}{n}$ where ϵ is 0.1. The simulation includes a data center with three hosts where each host is defined using specifications from SPEC benchmark. Up to five VMs are hosted with each VM having a maximum capacity of 10 GB RAM. For a simulation environment, this dataset represents a moderately scaled cloud infrastructure, providing a balanced mix of complexity and manageability.

Additionally, two additional datasets, $DS_{3}^{L}$ and $DS_{3}^{R}$, were generated as left-skewed and right-skewed variants of $DS_{3}$, respectively. These datasets were created using the same configuration parameters described in Section 4.1, with the task length distribution adjusted to introduce skewness. The left-skewed dataset ($DS_{3}^{L}$) emphasizes shorter task lengths, while the right-skewed dataset ($DS_{3}^{R}$) focuses on longer task lengths, providing insights on method’s performance on different workload conditions.

The parameter values in Table 5 were selected to simulate diverse scenarios representing small- to medium-sized cloud environments. A modest number of hosts and VMs were chosen to reflect realistic configurations often encountered in private or hybrid cloud setups, balancing computational capacity with practical constraints. Variation in the number of tasks and their dependencies (small, medium, and large DAGs) was introduced to capture diverse workflow complexities, ranging from simple workflows with few tasks to complex workflows with extensive interdependencies. Task characteristics, such as length, were varied across datasets to assess the scheduler’s adaptability to workloads with differing computational demands. Additionally, skewed datasets were generated by applying left and right skewness to the task lengths. This was conducted to introduce variability and test the robustness of the proposed model under scenarios where task distributions deviate from normal patterns, as might occur in dynamic real-world environments.

The proposed algorithm is compared against several baseline algorithms to comprehensively evaluate its performance and highlight its advantages. Table 6 summarizes the methods used, including HEFT, Min–Min, Max–Min, Round Robin, and Random. These algorithms were chosen not only for their established roles in task scheduling but also to provide a diverse set of perspectives on scheduling performance. HEFT, Min–Min, and Max–Min represent widely recognized heuristic strategies that excel in specific static or single-objective scenarios, making them valuable baselines for understanding fundamental scheduling behaviors. Round Robin and Random scheduling, while simplistic, serve as essential comparisons to underscore the significance of structured decision-making and dynamic adaptability. Though these algorithms may not match the sophistication of DRL-GNN in terms of dynamic adaptability or multi-objective optimization, their inclusion highlights the limitations of static and heuristic-based approaches, emphasizing the superior adaptability and versatility of DRL-GNN. By comparing against algorithms with varying levels of complexity, this study ensures a comprehensive evaluation, demonstrating how DRL-GNN consistently outperforms both rudimentary and heuristic-based methods across diverse scheduling scenarios.

Furthermore, this comparison aims to validate the effectiveness of GNN-based models in cloud workflow scheduling, demonstrating that a learning-based approach can successfully model task dependencies and dynamic resource allocations while achieving competitive scheduling performance. By illustrating how DRL-GNN outperforms traditional methods, this study establishes GNN-based Reinforcement Learning as a promising direction for future research in cloud workflow scheduling.

### 4.5. Result Analysis

**Figure 11:**
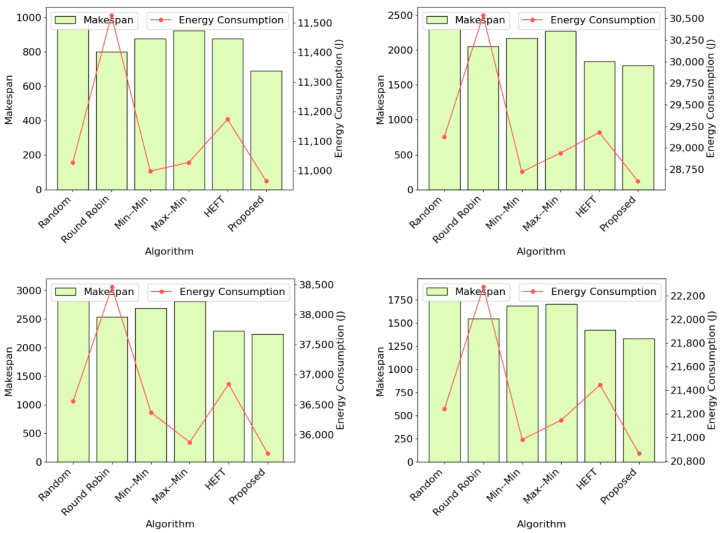
Comparison of makespan and energy consumption across algorithms for $DS_{2}$ (**top left**), $DS_{3}$ (**top right**), $DS_{3}^{L}$ (**bottom left**), and $DS_{3}^{R}$ (**bottom right**).

The performance of the proposed DRL-GNN framework was evaluated across three datasets ($DS_{1}$, $DS_{2}$, and $DS_{3}$), each representing varying levels of complexity and task concurrency. The results demonstrate consistent improvements in makespan and energy efficiency compared to baseline methods based on heuristics. The results and a detailed discussion of the findings are presented in Figure 11.

The results of the proposed algorithm are compared with the baseline methods described in Section 4. The evaluation metrics include makespan, active energy consumption, Energy-Delay Product (EDP), and Energy Per Task (EPT). Table 7 summarizes the performance of the proposed algorithm and baseline methods across these metrics. The table contains the average of 10 samples taken from the dataset parameters shown in Table 5.

In dataset ${\mathrm{DS}}_{1}$, which represents a low-concurrency environment with relatively small workflows, the proposed framework achieved a makespan of 194.71 s, surpassing the closest heuristic, HEFT, which recorded 201.87 s. This corresponds to a reduction of approximately 3.55% in makespan. In terms of active energy consumption, the proposed method achieved the lowest value of 3111.63 J, marginally outperforming Max–Min, which recorded 3125.44 J. These results underline the framework’s ability to provide balanced optimization in environments characterized by smaller workflows and limited task interdependencies.

For dataset ${\mathrm{DS}}_{2}$, which represents a moderately complex environment with larger workflows and increased variability, the proposed method demonstrated significant improvements in makespan and energy efficiency. It achieved a makespan of 689.22 s, which is a reduction of 13.92% compared to Round Robin, the closest competitor (800.72 s). Additionally, the framework recorded the lowest active energy consumption of 10,964.45 J, slightly better than Min–Min (10,998.52 J). These findings highlight the proposed framework’s effectiveness in handling moderately complex scheduling scenarios by leveraging its ability to model task dependencies and resource constraints dynamically.

In dataset ${\mathrm{DS}}_{3}$, characterized by a similar environment to ${\mathrm{DS}}_{2}$, but with a higher number of tasks inside a workflow (15–20 in $DS_{2}$ vs. 40–50 in $DS_{3}$), resulting in higher computational demands, the proposed framework exhibited robust performance. It achieved the lowest makespan of 1779.61 s, surpassing HEFT (1837.80 s) with an improvement of 3.17%. The proposed method recorded a slightly lower energy consumption (28,611.36 J) compared to Min-Min (28,720.47 J). The result of achieving a lower makespan while maintaining lower energy consumption demonstrates that the framework maintains competitive energy efficiency even in highly demanding scenarios. This result underscores the scalability and adaptability of the proposed method in dynamic and complex cloud environments.

In dataset ${\mathrm{DS}}_{3}^{L}$, with a left-skewed distribution favoring shorter tasks, and ${\mathrm{DS}}_{3}^{R}$, with a right-skewed distribution emphasizing longer tasks, the proposed framework consistently demonstrated superior performance across both scenarios. For ${\mathrm{DS}}_{3}^{L}$, the framework achieved the lowest makespan of 2238.99 s, outperforming HEFT (2293.07 s) by 2.36%, and recorded the lowest active energy consumption of 35,686.71 J, marginally surpassing Max–Min (35,872.85 J). In ${\mathrm{DS}}_{3}^{R}$, the proposed method excelled further with a makespan of 1330.26 s, a 6.45% improvement over HEFT (1421.96 s), and the lowest energy consumption of 20,864.43 J, narrowly outperforming Min–Min (20,981.35 J). These results underscore the framework’s adaptability, effectively optimizing makespan and energy consumption across diverse workload distributions in dynamic cloud environments with varying workloads.

To complement the discussion of results, EDP and EPT metrics provide deeper insights into the trade-offs between makespan reduction and energy efficiency. The EDP, calculated as the product of active energy consumption and makespan, highlights the cumulative energy cost associated with achieving faster task completion times. A lower EDP indicates a better balance between time and energy efficiency. Similarly, the EPT metric normalizes energy consumption over the number of tasks, offering a task-level perspective on energy efficiency. Across datasets, the proposed method consistently achieves the lowest EDP and EPT values, demonstrating its ability to optimize energy consumption while maintaining competitive makespan performance. These metrics reinforce the multi-objective effectiveness of the proposed algorithm.

### 4.6. Algorithm Runtime Analysis

The runtime of the proposed algorithm is slightly higher compared to heuristic-based approaches; however, it does not exhibit exponential growth. Unlike batch-based heuristic algorithms that compute scheduling solutions in a single step, the proposed approach operates as an online scheduling algorithm, making scheduling decisions iteratively. Consequently, while the total runtime is relatively high, the per-decision latency remains low. Since each decision in the scheduling process is made via inference using a trained reinforcement learning model, the computational cost per decision remains constant, primarily determined by the number of parameters in the model. This ensures scalability even as task complexity grows.

**Figure 12:**
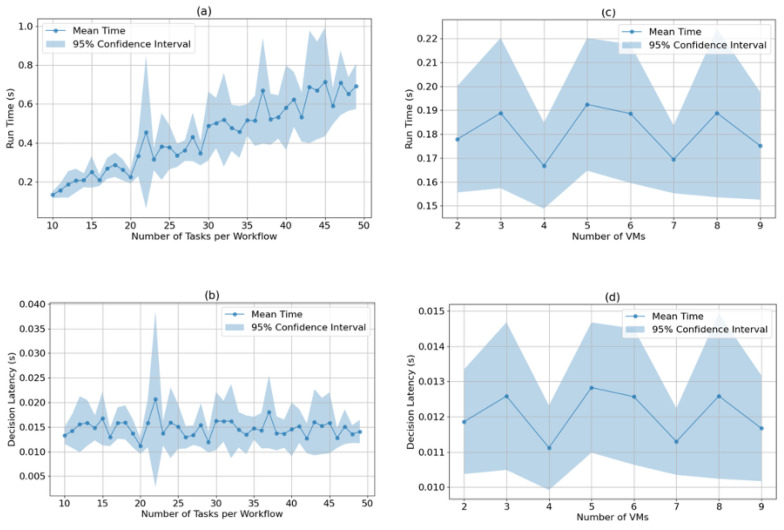
Runtime and decision latency variation with different parameters: (**a**) Runtime vs. number of tasks per workflow, (**b**) Decision latency vs. number of tasks per workflow, (**c**) Runtime vs. number of VMs, and (**d**) Decision latency vs. number of VMs.

To further analyze the performance, runtime of the algorithm across various scenarios was plotted as per Figure 12. As the number of tasks per workflow increases, the total runtime increases in a linear fashion as shown in Figure 12a,b, while the decision latency remains somewhat constant. As the number of VMs increases, the runtime does not change drastically as indicated in Figure 12c,d. This behavior is explained by the fact that the number of tasks influences the total number of decisions the algorithm must make, which in turn affects total runtime. However, the time required per decision remains low, making the proposed approach suitable for real-time and dynamic scheduling scenarios.

### 4.7. Statistical Analysis

Table 8 provides the results of statistical tests conducted to evaluate the performance of different scheduling algorithms across three datasets ($DS_{1}$, $DS_{2}$, and $DS_{3}$) for two key metrics: makespan and energy. The table includes the results of the Friedman test, a non-parametric statistical test used to detect overall differences among the algorithms, followed by Wilcoxon signed-rank tests, which perform pairwise comparisons between the proposed algorithm and other baseline algorithms to determine if the observed differences are statistically significant.

For makespan, the Friedman test indicates statistically significant differences among the algorithms across all datasets (*p*-value = 0.0000). The pairwise Wilcoxon tests show that the proposed algorithm consistently outperforms all other methods, with statistically significant improvements (*p*-value < 0.05) against Random, Round Robin, Min–Min, and Max–Min across all datasets. The results for HEFT, while better than other baselines in some cases, show non-significant differences (*p*-value > 0.05) for $DS_{1}$ and $DS_{3}$, indicating comparable performance in certain scenarios. For energy, the Friedman test results vary across datasets. For $DS_{1}$, no statistically significant overall differences are detected among the algorithms (*p*-value = 0.0619). However, for $DS_{2}$ and $DS_{3}$, significant differences are observed (*p*-value < 0.05). Pairwise Wilcoxon tests highlight significant energy efficiency improvements for the proposed algorithm compared to Random and Round Robin methods across $DS_{2}$ and $DS_{3}$. Against Min–Min, Max–Min, and HEFT, the differences are less pronounced, with several non-significant results, particularly in $DS_{1}$. These findings suggest that while the proposed method improves energy efficiency, the magnitude of the improvement depends on the complexity and scale of the dataset.

### 4.8. Multi-Objective Solution Analysis

**Figure 13:**
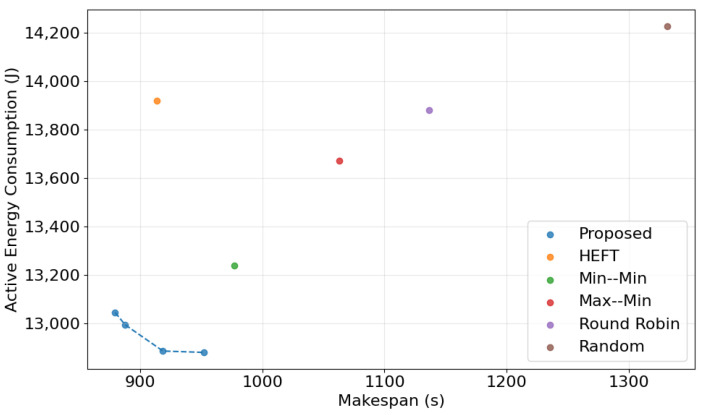
Pareto front illustrating the trade-offs between makespan and active energy consumption achieved by the proposed DRL-GNN framework compared to baseline algorithms.

To evaluate the multi-objective optimization capabilities of the proposed DRL-GNN framework, a Pareto front diagram was generated and is presented in Figure 13. The Pareto front illustrates the trade-offs between makespan and active energy consumption for the proposed method and baseline algorithms. The proposed DRL-GNN framework achieves a superior trade-off. In comparison, baseline methods such as HEFT, Min–Min, Max–Min, Random, and Round Robin are scattered further from the Pareto front, reflecting their limitations in optimizing both objectives simultaneously. While HEFT and Min–Min provide relatively competitive solutions, their points remain dominated by the proposed method, which consistently outperforms them in both makespan and energy consumption. These findings highlight the ability of the proposed framework to deliver dependency-aware, dynamic scheduling decisions while effectively balancing multiple objectives.

When compared against a multi-objective scheduling algorithm such as MOHEFT [49], the performance of the proposed DRL-GNN framework was analyzed using two widely recognized multi-objective evaluation metrics, hypervolume and Inverted Generational Distance (IGD). The hypervolume metric measures the volume in the objective space that is dominated by the obtained Pareto front relative to a predefined reference point, with a higher hypervolume indicating a better approximation of the true Pareto front [50]. IGD, on the other hand, quantifies the distance between the obtained Pareto front and a reference set of optimal trade-off solutions, with lower IGD values indicating better diversity and proximity to the optimal front [50].

The results indicate that while the proposed method achieves better makespan reduction, its obtained solutions exhibit a less diverse distribution compared to MOHEFT, as evidenced by the lower hypervolume and higher IGD values (Table 9). This suggests that the model, despite optimizing makespan and energy consumption simultaneously, lacks the flexibility to generate a broad range of trade-off solutions. The reduced diversity can be attributed to the fixed nature of the reward function, which does not allow for dynamically adjusting the importance of makespan versus energy consumption during scheduling. As a result, the learned policy tends to converge towards a specific balance, rather than exploring a wider range of potential Pareto-optimal solutions.

**Figure 14:**
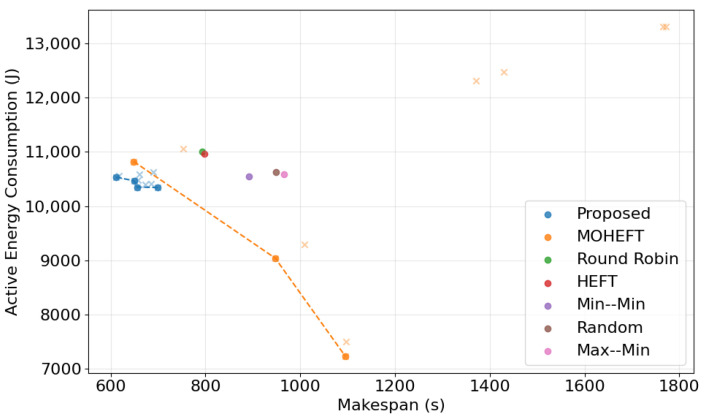
Pareto front illustrating the trade-offs between makespan and active energy consumption achieved by the proposed DRL-GNN framework compared to baseline algorithms and MOHEFT algorithm.

To address this limitation, future work will focus on enhancing the adaptability of the DRL-GNN framework by incorporating an adjustable weighting mechanism for reward functions. Instead of using fixed weights for makespan and energy consumption, the model could take these weights as an input, allowing for user-defined priorities and generating a more diverse set of scheduling solutions. This approach would enable the framework to produce Pareto fronts with greater flexibility, adapting to varying optimization objectives as required by different cloud workflow scenarios. Figure 14 illustrates the limited diversity of the proposed framework’s Pareto front compared to MOHEFT, further supporting the need for improved adaptability in future iterations.

### 4.9. Analysis Summary

Overall, the results indicate that the proposed DRL-GNN framework consistently outperforms traditional heuristic methods, demonstrating its effectiveness in achieving multi-objective optimization by minimizing makespan and reducing energy consumption across datasets of varying complexity. The consistent statistical significance in pairwise comparisons underscores its robust performance, particularly in complex scheduling scenarios ($DS_{2}$ and $DS_{3}$). The results also suggest that the advantage in energy optimization is more nuanced, as some baselines perform similarly in less demanding environments. While the improvements in energy efficiency are sometimes modest, the framework’s capability to balance multiple objectives, including makespan minimization and energy efficiency, highlights its potential as a viable solution for dynamic task scheduling in cloud computing environments. The relatively smaller gains in energy consumption compared to makespan reductions can be attributed to the inherent trade-offs in task scheduling. For instance, optimizing for a shorter makespan often requires utilizing more computational resources simultaneously, which can lead to higher power consumption despite improved efficiency. Nevertheless, the framework’s ability to address task dependencies and resource constraints effectively, while still maintaining competitive energy efficiency, makes it particularly suitable for workflows with diverse computational requirements and dynamic cloud environments.

## 5. Discussion

This study introduces several novel contributions compared to prior work in the domain of cloud workflow scheduling. Firstly, the proposed method supports workflows in the form of DAGs which are captured by GNNs with encoded task and VM states, enabling the model to capture relationships within the workflow’s DAG. Secondly, an immediate reward structure, grounded in baseline lower-bound heuristics, is employed instead of relying solely on terminal rewards. Thirdly, the framework demonstrates adaptability to dynamic tasks and VMs by training on one VM count/host count configuration and successfully generalizing to diverse configurations. These key distinctions are summarized in Table 10, alongside comparisons with existing studies.

The method adapts to resource variability by leveraging the RL interaction loop, which continuously samples the observation space, including workflow structures and VM configurations, for each scheduling decision. Unlike static algorithms, this approach ensures that changes in resources are reflected in the results dynamically. Furthermore, the RL model, trained on one set of configurations, has demonstrated the ability to generalize solutions across configurations within a similar range, as shown in the evaluation section. This adaptability allows the scheduler to respond effectively to dynamic changes in resource availability and workload demands. However, in the current implementation, the scheduler requires a restart to detect new changes in the environment. Future work could focus on enabling real-time detection of resource changes to further enhance the adaptability of the scheduling method.

While the framework has shown promise, certain limitations were observed. The improvements in energy efficiency, although evident, were not uniformly significant across all scenarios, highlighting the need for further refinement in reward function design. Additionally, even though the inference time for one action is mostly constant, the computational complexity of the training process may pose challenges for scaling the approach to larger datasets or infrastructure setups.

While the evaluation setup mentioned in Section 4.4 captures essential heterogeneity in CPU speeds, RAM allocations, and task lengths, it also simplifies certain aspects of real-world cloud environments for initial experimentation. Large-scale cloud systems, which often involve thousands of VMs, dynamic resource provisioning, and geographically distributed data centers, introduce additional complexities that can be explored in future studies. Future work can aim to bridge this gap by scaling the simulation environment to more closely resemble real-world cloud scenarios. This can include increasing the number of hosts, VMs, and tasks to match the scale of modern cloud data centers, as well as incorporating dynamic workload variations, network latencies, and energy-aware configurations. These extensions can enable a more comprehensive evaluation of the proposed algorithm’s robustness and adaptability, ensuring its applicability to the demands of real-world cloud computing environments.

Moreover, the results indicate that while the proposed framework optimizes both makespan and energy consumption, it lacks the flexibility to generate a diverse set of trade-off solutions. This limitation stems from the fixed reward function, which does not allow for dynamic prioritization of objectives. Future work could focus on introducing an adjustable weighting mechanism, where the model takes makespan and energy consumption weights as inputs. This would enable adaptive scheduling strategies, allowing the framework to generate a broader range of Pareto-optimal solutions tailored to different user requirements. By doing so, the model could provide greater flexibility in balancing optimization objectives across varying cloud workflow scenarios. Further, investigating advanced exploration strategies in Reinforcement Learning, such as reward shaping or policy regularization, could improve the model’s ability to explore diverse scheduling solutions more effectively.

Future work could also explore the incorporation of more energy-specific reward functions, such as those prioritizing energy-aware VM allocation. Alternative optimization objectives, such as service-level agreement (SLA) adherence, could be investigated to complement the current study’s focus on makespan and energy consumption. Real-world datasets would provide a valuable avenue for validating the method’s applicability in practical settings. Furthermore, an exploration of hierarchical agents that use this approach but in a hierarchical manner (one agent choosing the task to run, while the other chooses the VM) would be beneficial to explore. Additionally, future studies could focus on an in-depth comparison of the proposed framework with other state-of-the-art (SOTA) algorithms to better understand its relative performance in various scheduling scenarios and further establish its competitiveness.

## 6. Conclusions

This paper proposed a novel framework for dynamic workflow scheduling in cloud environments by integrating Graph Neural Networks with Deep Reinforcement Learning using the Proximal Policy Optimization algorithm. The framework aimed to optimize two key objectives within a multi-objective optimization framework: minimizing makespan and reducing energy consumption. Experimental results in diverse datasets demonstrated that the proposed method achieves marginal but consistent improvements over traditional heuristic approaches, particularly in scenarios that involve dynamic workloads and task dependencies. The study contributes to the field of cloud resource management by addressing the challenges of dynamic scheduling with dependent tasks. Future work will focus on improving energy optimization, evaluating the framework in real-world settings, and exploring alternative RL approaches to improve both scalability and performance.

## Figures and Tables

**Table 1:** Summary of input features.

Feature		Name	Description
$F_{i,t}^{\mathrm{ts}}$	$I_{J_{i},t}^{\mathrm{sch}}$	Task-scheduled flag	A binary flag indicating whether the task has already been scheduled.
$F_{i,t}^{\mathrm{tr}}$	$I_{J_{i},t}^{\mathrm{ready}}$	Task-ready flag	A binary flag denoting whether the task is ready to be scheduled, determined by checking if all its parent tasks are scheduled.
$F_{i,t}^{\mathrm{tc}}$	$M_{J_{i},t}^{\mathrm{est}}$	Task completion time	The estimated completion time for unscheduled tasks, or the actual completion time for tasks that are already scheduled. (Equation (11))
$F_{i,t}^{\mathrm{vmc}}$	$M_{{\mathrm{VM}}_{i},t}$	VM completion time	The time at which the VM will become available for scheduling new tasks, based on the currently scheduled tasks. (Equation (12))
$F_{i,t}^{\mathrm{vms}}$	$\frac{1}{S_{{\mathrm{VM}}_{i},t}}$	VM CPU speed	The inverse of CPU speed of the VM in MIPS. The inverse is used to improve the model convergence.
$F_{i,t}^{\mathrm{vme}}$	$\frac{P_{H_{k}}^{\mathrm{peak}}-P_{H_{k}}^{\mathrm{idle}}}{S_{H_{k}}}$	VM active energy consumption rate	The active energy consumption of a VM, calculated using the host characteristics ($H_{k}$ being the host of ${\mathrm{VM}}_{i}$).
$A_{i,j,t}$	$I_{J_{i},{\mathrm{VM}}_{j},t}^{\mathrm{cmp}}$	Compatibility flag	A binary flag indicating whether the task can be executed on the VM, based on the task’s resource requirements and the VM’s specifications ($R_{{\mathrm{VM}}_{j}}\geq R_{J_{i}}$).
$B_{i,j,t}$	$I_{J_{i},J_{j},t}^{\mathrm{dep}}$	Task dependency flag	A binary flag representing whether there is a dependency (directed edge) from task $J_{i}$ to task $J_{j}$. If a task is scheduled to a VM, a new dependency is added between the previous task of the VM and the scheduled task.

**Table 2:** Host characteristics as taken from SPECpower_ssj^®^ 2008 benchmark suite [6].

Name	Number of Cores	CPU Speed (GIPS)	RAM (GB)	Idle Power (W)	Peak Power (W)
PowerEdge R740	56	604.8	64	50	432
PowerEdge C6100	48	587.52	64	227	895
PowerEdge R820	32	332.8	64	110	446
iDataPlex dx360	16	187.712	48	116	475
PowerEdge R710	12	146.88	64	62	227

**Table 3:** Training dataset generation parameters.

Parameter	Description	Value
$N^{H}$	Number of hosts	4
$N^{\mathrm{VM}}$	Number of VMs	10
$N^{W}$	Number of workflows	10
$N^{J}$	Number of tasks in a workflow	20
$S_{\mathrm{VM}}$	CPU speed of a VM in MIPS	[500, 5000]
*R*	Amount of RAM for a VM (in GB)	[1, 10]
*L*	Task length	[500, 100,000]

**Table 4:** Hyperparameters used for PPO training.

Parameter	Description	Value
lr	Learning rate	$2.5\times 10^{-4}$
*N*	Batch size	512
γ	Discount factor	0.99
λ	Generalized Advantage Estimation (GAE) factor	0.95
ϵ	Clipping parameter	0.2
*K*	Number of epochs per PPO update	4
β	Entropy coefficient	0.01
*c*	Maximum gradient norm for clipping	0.5
*B*	Number of steps per policy rollout	128
*M*	Number of minibatches per update	4
$\theta_{\mathrm{je}}$	Job encoder hidden layers	[32,32]
$\theta_{\mathrm{me}}$	Machine encoder hidden layers	[32]
$\theta_{\mathrm{ee}}$	Edge encoder hidden layers	[32]
$\theta_{\mathrm{ee}}$	Edge scorer hidden layers	[64,32]
$\theta_{\mathrm{ee}}$	Graph scorer hidden layers	[32,32]
*E*	Number of features in embeddings	32
$L_{G}$	Number of GIN layers	3
α	Weight of makespan reward	1
β	Weight of energy consumption reward	1

**Table 5:** Evaluation dataset generation parameters.

Parameter	Description	${\mathrm{DS}}_{1}$	${\mathrm{DS}}_{2}$	${\mathrm{DS}}_{3}$
$N^{H}$	Number of hosts	3	3	3
$N^{\mathrm{VM}}$	Number of VMs	4	5	5
$N^{W}$	Number of workflows	10	10	10
$N^{J}$	Number of tasks in a workflow	[1,5]	[15,20]	[40,50]
$S_{\mathrm{VM}}$	CPU speed of a VM in MIPS	[2500,5000]	[1000,5000]	[1000,5000]
*R*	Amount of RAM for a VM	[1,2]	[1,10]	[1,10]
*L*	Task length	[50,000, 100,000]	[500, 100,000]	[500, 1,000,000]

**Table 6:** Summary of comparison algorithms.

Algorithm	Description
Random	Randomly assigns tasks to VMs.
Round Robin [24]	Cyclically assigns tasks to VMs.
Min–Min [24]	Assigns tasks with the shortest execution time to the VM that completes them the fastest.
Max–Min [24]	Assigns tasks with the longest execution time to the VM with the earliest completion time.
HEFT [24]	Schedules workflows individually using the Heterogeneous Earliest Finish Time algorithm.

**Table 7:** Performance comparison of the proposed algorithm and baselines.

Dataset	Algorithm	Makespan (s)	Active Energy Consumption (J)	EDP (Js) $E_{\mathrm{total}}\times M$	EPT (J) $\frac{E_{\mathrm{active}}}{N^{\mathrm{Tasks}}}$	Decision Latency (s)	Total Run Time (s)
${\mathrm{DS}}_{1}$	Random	306.61	3225.12	130,782,928.46	103.39	0.01	0.01
	Round Robin	246.39	3140.78	105,123,167.65	100.65	0.01	0.01
	Max–Min	210.27	3125.44	89,671,938.18	100.26	0.01	0.01
	Min–Min	206.54	3128.09	88,041,738.35	100.38	0.01	0.01
	HEFT	201.87	3138.89	85,887,677.23	100.59	0.01	0.01
	Proposed	194.71	3111.63	82,751,239.30	99.80	0.01	0.19
${\mathrm{DS}}_{2}$	Random	1009.21	11,027.32	962,195,838.37	63.15	0.01	0.01
	Round Robin	800.72	11,525.60	764,351,107.50	66.02	0.01	0.01
	Max–Min	924.37	11,028.04	880,585,573.80	63.15	0.01	0.01
	Min–Min	876.27	10,998.52	836,079,990.97	63.00	0.01	0.01
	HEFT	876.81	11,173.97	835,583,187.95	63.99	0.08	0.08
	Proposed	689.22	10,964.45	657,333,112.54	62.79	0.01	2.50
${\mathrm{DS}}_{3}$	Random	2494.47	29,127.95	5,294,475,677.89	64.20	0.01	0.01
	Round Robin	2056.44	30,536.36	4,366,303,181.27	67.3	0.01	0.01
	Max–Min	2272.08	28,939.87	4,819,828,319.41	63.79	0.01	0.01
	Min–Min	2170.36	28,720.47	4,604,694,515.36	63.32	0.01	0.01
	HEFT	1837.80	29,178.38	3,899,255,596.67	64.32	0.36	0.36
	Proposed	1779.61	28,611.36	3,774,081,849.42	63.08	0.05	21.32
${\mathrm{DS}}_{3}^{L}$	Random	3051.34	36,554.21	7,893,707,255.36	81.36	0.02	0.02
	Round Robin	2539.95	38,456.79	6,575,378,599.92	85.60	0.02	0.02
	Max–Min	2808.50	35,872.85	7,266,347,878.71	79.85	0.02	0.02
	Min–Min	2685.12	36,370.58	6,948,309,882.74	80.96	0.02	0.02
	HEFT	2293.07	36,842.30	5,932,778,716.71	82.01	0.34	0.34
	Proposed	2238.99	35,686.71	5,792,398,179.82	79.43	0.04	20.12
${\mathrm{DS}}_{3}^{R}$	Random	1883.77	21,241.13	3,071,269,569.34	47.49	0.03	0.03
	Round Robin	1548.01	22,274.42	2,526,040,425.56	49.80	0.03	0.03
	Max–Min	1704.66	21,146.16	2,779,509,096.67	47.27	0.03	0.03
	Min–Min	1683.71	20,981.35	2,745,459,751.10	46.91	0.03	0.03
	HEFT	1421.96	21,444.84	2,318,718,160.47	47.95	0.41	0.41
	Proposed	1330.26	20,864.43	2,169,170,282.82	46.65	0.05	23.85

**Table 8:** Statistical test results for scheduling algorithms across datasets.

Metric	Dataset	Friedman Stat	*p*-Value	Compared Algorithm (vs. Proposed)	Wilcoxon Stat	*p*-Value
Makespan	$DS_{1}$	38.00	0.0000	Random	0.00	0.0020
				Round Robin	0.00	0.0020
				Min–Min	7.00	0.0371
				Max–Min	7.00	0.0371
				HEFT	15.00	0.2324
	$DS_{2}$	41.03	0.0000	Random	0.00	0.0020
				Round Robin	0.00	0.0020
				Min–Min	0.00	0.0020
				Max–Min	0.00	0.0020
				HEFT	0.00	0.0020
	$DS_{3}$	44.63	0.0000	Random	0.00	0.0020
				Round Robin	0.00	0.0020
				Min–Min	0.00	0.0020
				Max–Min	0.00	0.0020
				HEFT	10.00	0.0840
Energy	$DS_{1}$	10.51	0.0619	Random	3.00	0.0098
				Round Robin	11.00	0.1055
				Min–Min	19.00	0.4316
				Max–Min	23.00	0.6953
				HEFT	22.00	0.6250
	$DS_{2}$	25.14	0.0001	Random	26.00	0.9219
				Round Robin	0.00	0.0020
				Min–Min	25.00	0.8457
				Max–Min	21.00	0.5566
				HEFT	3.00	0.0098
	$DS_{3}$	26.06	0.0001	Random	3.00	0.0098
				Round Robin	0.00	0.0020
				Min–Min	23.00	0.6953
				Max–Min	14.00	0.1934
				HEFT	1.00	0.0039

**Table 9:** Performance and solution comparison of the proposed algorithm and MOHEFT.

Dataset	Algorithm	Makespan (s)	Active Energy Consumption (J)	Hypervolume	IGD	Decision Latency (s)	Total Run Time (s)
${\mathrm{DS}}_{1}$	MOHEFT	428.35	2758.44	1,274,777.84	2458.88	0.02	0.02
	Proposed	194.71	3111.63	983,181.54	2869.48	0.01	0.19
${\mathrm{DS}}_{2}$	MOHEFT	1234.29	10,096.99	7,748,022.07	7486.76	0.14	0.14
	Proposed	689.22	10,964.45	4,804,159.93	10,928.23	0.01	2.50
${\mathrm{DS}}_{3}$	MOHEFT	3245.56	26,123.24	7,748,022.07	18,594.15	0.02	0.02
	Proposed	1779.61	28,611.36	4,804,159.93	27,340.00	0.05	21.32

**Table 10:** Comparison of proposed algorithm with existing approaches.

Algorithm	Task Dependencies	Reward	Objectives	Simulation Tool	Dataset Type	Compared Algorithms
DRL-Cloud [31]	Yes	Terminal	Energy, Cost	Custom (Python, TensorFlow)	Synthetic *	Greedy, FERPTS, Round Robin
DRLBTSA [32]	No	Terminal	Energy, SLA Violations, Makespan	CloudSim	Synthetic *	Round Robin, FCFS, Earliest Deadline First, RATS-HM, MOABCQ
HunterPlus [33]	No	Terminal	Makespan, Energy	COSCO	Synthetic	GGCN, Bidirectional GGCN
DeepRM Plus [34]	No	Terminal	Turnaround Time, Cycling Time	Custom (Python, TensorFlow)	Real-World	Random, FCFS, SJF, HRRN, Tetris, DeepRM
MADRL [11]	Yes	Terminal	Energy Efficiency, Time	CloudSim	Synthetic	Random, Greedy, Common-Actor
DPSO-GA [35]	No	Terminal	Waiting Time, Task Migration, Response Time, Task Running Time	CloudSim	Real-World	GA, PSO
Proposed Algorithm	Yes	Immediate	Makespan, Energy Consumption	CloudSim	Synthetic*	Random, Round Robin, Min-Max, Min–Min, HEFT

* Mostly synthetic dataset with some information such as hosts or VMs derived from real-world datasets or datasheets.

## Data Availability

SPECpower_ssj 2008 Results https://www.spec.org/power_ssj2008/results/ (accessed on 1 September 2024). Source code available at https://github.com/kdsuneraavinash/workflow-cloudsim-drlgnn (accessed on 25 December 2024).
